# Back to the Future of Quantitative Psychology and Measurement: Psychometrics in the Twenty-First Century

**DOI:** 10.3389/fpsyg.2017.02099

**Published:** 2017-12-01

**Authors:** Pietro Cipresso, Jason C. Immekus

**Affiliations:** ^1^Applied Technology for Neuro-Psychology Lab, Istituto Auxologico Italiano, Milan, Italy; ^2^Department of Psychology, Catholic University of the Sacred Heart, Milan, Italy; ^3^Department of Educational Leadership, Evaluation and Organizational Development, College of Education and Human Development, University of Louisville, Louisville, KY, United States

**Keywords:** quantitative psychology, measurement, psychometrics, computational psychometrics, mathematical psychology

Measurements in psychology always have been a significant challenge. Research in quantitative psychology has developed several methods and techniques to improve our understanding of humans. Over the last few decades, the rapid advancement of technology had led to more extensive study of human cognition, including both the emotional and behavioral aspects. Psychometric methods have integrated very advanced mathematical and statistical techniques into the analyses, and in our Frontiers Specialty (Quantitative Psychology and Measurement), we have stressed the methodological dimension of the best practice in psychology. The long tradition of using self-reported questionnaires is still of high interest, but it is not enough in the twenty-first century.

We stress the use of innovative methods and technologies as psychometric tools. One of the most significant challenges in quantitative psychology and measurement concerns the integration of technologies and computational techniques into current standards.

In the following, our aim is to show how data collection can involve human behavior, internal states and the manipulation of experimental settings. In particular, we define typical psychophysiological measures for a deeper understanding of internal states—analyzing the central and peripheral nervous system, hormonal factors in the endocrine system and the fascinating field of gene transcription in human neuroscience. These factors represent the measurement of the “internal” sphere that is becoming so interesting for measurement in all the field of psychology, including social and affective science, not only in the cognitive sciences. The idea to read internal states has always been very clear in clinical and experimental psychology, but now is becoming even more widespread. This is thanks to the improvements in technologies and lower costs.

Next, we highlight the measurement of the exhibited behavior patterns representing the “external” sphere of human thinking through expressed behavior. Again, technology is a critical aspect shedding new light on the field. The use of low-cost and high-end technologies for understanding verbal and nonverbal patterns is helping to identify innovative ways to measure the psychological factors leading to a behavior. They can be considered a new challenge of behavioral science, e.g., the use of commercial devices (such as the Kinect) in motor and cognitive neurorehabilitation. Linked to psychophysiology and exhibited behavior patterns, virtual reality is becoming a cutting-edge tool for experimental manipulation, building personalized experimental settings, but found in a laboratory.

We define and highlight the use of virtual reality in psychology as an incredible low-cost tool collecting data and creating realistic situations that can be used for clinical, experimental, social settings among others, and so of keen interest in several psychology fields.

In conclusion, we present new methods and techniques already used in other fields, but incredibly expanding also in psychology and psychometrics. Computational science, complex networks, and simulations, are highlighted as the promising new methods for the best convergence of psychological science and technologies. These have ability to create innovative tools for better comprehension and a quantitative measurement in psychology.

## Psychophysiology: nervous system, endocrine system, and gene transcription

The use of biosensors in human research has become a reliable method for a quantitative and objective measurement of participants' at psychological, behavioral, and physiological level. The use of biosensors, specifically psychophysiology, is not an alternative to self-reports, but they can be considered as a great asset in our effort to integrate additional information to enhance our understanding of specific patterns.

The advantage of psychophysiology is the possibility of recording internal states during an experience (Mauri et al., 2010; Blascovich, 2014; Kreibig et al., 2015; McGaugh, 2016). This means that the researcher can evaluate the impact of a specific experience without interrupting the experience to ask the user her/his opinion.

In the valence-arousal model (Russell, 1979; Lang, 1995) researchers are interested in the identification of affective states of subjects during experimental sessions. There exists two “activation” dimensions mainly investigated, namely the physiological arousal and emotional valence. The Arousal-Pleasantness plan is drown in psychophysiology as a robust measurement of the affective states. Physiological arousal is a measure of the sympathetic branch of the autonomic nervous system (ANS). Sympathetic activation generates an increase in the activity of sudoriparous glands (also known as Sweat glands) that it is possible to measure trough Electrodermal activity (EDA) computed as galvanic skin response (GSR), or also as skin conductance resistance (SCR). EDA is a direct measure of sympathetic activation with no intervention of the parasympathetic branch. Other good measures of physiological arousal that are affected by the two branches of ANS are skin temperature, heart rate, respiration rate, and pupil dilation. To measure emotional valance is more complex, and researchers generally used facial expression for identifying this dimension. At psychophysiological level facial expressions have been tested through the use of superficial electromyography (sEMG) a noninvasive way to quantify the muscle activation. In particular activations of the zygomatic major and corrugator supercilii facial muscles are known as the best indicators of emotional valence. We will have a positive valence with increased activity of the zygomatic major muscle, and a negative valence with a higher activation of corrugator supercilii facial muscle (Blumenthal et al., 2005). Since emotional valence identify positive or negative direction we might be interested in a more direct measure of emotional intensity, and the best index to this purpose is the pupil dilation (Mauri et al., 2010).

Respiratory activity can be recorded to identify both the voluntary and the autonomic respiration activity on cardiovascular activity, that can also be recorded. In particular respiration (RSP) can be recorded through respiratory inductance plethysmography (RIP) with thoracic and abdominal strips. On the other hand cardiac activity can be recorded through an electrocardiogram (ECG), and in particular identifying the R peak in the ECG waveform (with the conventional PQRSTU peaks). The oscillations in R to R peaks in the ECG waveform (also known as NN intervals to emphasize normal beats) provide several information on sympathovagal activations and can be used to compute heart rate variability (HRV) indexes in the temporal and spectral domain. According to the guidelines of the Task Force of the European Society of Cardiology and the North American Society of Pacing and Electrophysiology, temporal, spectral and nonlinear indexes of HRV, can be considered as a robust way to identify responses in the ANS (Malik, 1996).

Electroencephalographic (EEG) analyses of both the classic spectral bands (e.g., alpha, beta, delta, gamma, …) and the related potential (ERP) that is evoked also have been used extensively in psychophysiological research (Thompson, 2016). More in general the data collection related to the brain is one of the huge field in neuroscience and psychology. In particular neuroimaging techniques received a lot of attention in psychological science and are able to provide a wide spectra of information related to the human thinking related to cognitive, affective and relational aspects (Cipresso et al., 2012b). Moreover, the improvement in the quality of the used methods to automatize the analysis of brain imaging results, provided new access to important information of structural brain and the related connections. This has been made possible by using deep learning and computational techniques, but also integrating different kinds of methods, such as EEG in the scanner addressing both spatial and temporal precision, and the interesting development of PET and fMRI, including spectroscopy, diffusion, connectome and the other challenges that neuroimaging and neuroscience methods are able to provide.

By using biosensors to record peripheral and central nervous activity, we can obtain an incredibly huge amount of data that can be analyzed for a deeper understanding of internal states during experimental tasks in psychological studies. In any case there are also several problems with the use of biosensors, in particular the obtrusiveness needs to be considered. Even if the goal is to not interfere with the experiment, this cannot be avoided and in a way or another we finish to affect behavior by measuring them. In most of the behavioral experiments, i.e., research related to emotions, affective states, and cognitive assessment, conditioning the participants is an important part that can risk to affect the validity and the reliability of a study.

This is a well-known problem in all the fields of research and there are different ways to deal with this issue. First, more precisely, less-intrusive biosensors have been developed. Also, there have been significant advancements in the development of integrated biosensors with emphasis to avoid any discomfort, building wearable biosensors without patches or cables.

Human experiments necessarily involve technologies used to read the impact of an experience goal in the study. However, the use of these technologies is not transparent to the participants and the more we want to know, the more will be investigated with sensors that will be evident in the subject. We need to seriously wonder if there is a way to have contactless biosensors, able to collect data which remains invisible to the data generator, that is a human being. In physics, scientists need to face the same problem which is well-known as the “observer effect,” where a physical measurement of a system is possible only influencing the system itself. In biobehavioral sciences the observer effect is even worse (Lewandowski et al., 2016). In consciousness research, the observer effect can be circumvented through “no-report” paradigms, where the idea is precisely to avoid asking people to report on their experiences to avoid the observer effect.

Interestingly, a foundational part of psychology and psychometrics consists not only of observing one's experiences, but also to think about them and report them. This appears to be a keen question where we are called to provide feedback as researchers in the field. Pervasiveness and ubiquitous computing could be a field that could provide some solutions by using biosensors integrated in technology—so personal as to be as invisible to individuals.

Considering smartphones and the data collected by the integrated sensors (such us the interconnected heart rate watches), we can understand that data collection can be transparent to everyone. In a big data world, it probably is easier to infer information from existing devices than collecting new data. On the other hand, this requires a change in knowledge and methods, i.e., to change from laboratory experimental-driven designs to field data-driven analyses. Even if this scenario is possible, it is not the solution to each measurement problems, but still a useful integration and direction to pursue the best way for a better understanding.

Psychophysiology addresses both the activities of the nervous system and the endocrine system, i.e., the collection of glands in people that secrete hormones directly into the circulatory system to reach distant target organs. Concentrations of hormones, patterns of released hormones, and the numbers and locations of the receptors of the hormones are related significantly with human behavior at the cognitive, emotional, and relational levels. Moreover, the efficiency of hormone receptors is involved in gene transcription and vice versa. In particular, hormones influence human behavior, which in turn can influence hormones, and the cycle continues. Thus, the endocrine system also must be investigated as an important measurement in psychology. For example, several studies about psychological stress demonstrated that prolonged stress causes the release of glucocorticoid (Lazzarino et al., 2013; Cattaneo and Riva, 2016). This release is controlled by the hypothalamus (such as the sympathetic nervous system, which is activated by acute, time-limited stressors), but, in this case, the control is endocrinal. In fact, the hypothalamus, with a release factor [corticotropin-releasing hormone (CRH)], induces the pituitary gland to release adrenocorticotropic hormone (ACTH), also known as corticotropin, that targets the adrenal glands (also known as suprarenal glands) (Popoli et al., 2012; Fries et al., 2016). This process, referred to as the hypothalamic–pituitary–adrenal axis (HPA axis or HTPA axis), is a major neuroendocrine system that manages stress reaction regulating several body functions (among which, digestion, emotions, and sexuality) (Dickerson and Kemeny, 2004). The most important glucocorticoid is cortisol, which is indeed considered to provide an objective measure of chronic stress. The metabolic effect of cortisol is slower than the effect of adrenaline, but it lasts longer (Singh et al., 1999). The release of cortisol has several effects on an organism, such as increasing the serum glucose with gluconeogenesis, increasing the metabolism of fat, and suppressing the immune system (to save energy). In addition, it shows negative effects on some cognitive processes, such as memory and attention (McEwen and Sapolsky, 1995) by affecting the hippocampus, which mediates the cortisol-induced feedback inhibition of the HPA axis. Cortisol also can produce the death of neural cells in the frontal lobe as well as producing detrimental consequences on the cardiovascular apparatus (Ockenfels et al., 1995; Miller et al., 2007).

## Analysis of exhibited behavior patterns

Gomez-Marin and colleagues (Gomez-Marin et al., 2014; Gomez-Marin and Mainen, 2016) defined animal behavior as “the macroscopic expression of neural activity, implemented by muscular and glandular contractions acting on the body, and resulting in egocentric and allocentric changes in an organized temporal sequence” (p. 1456). Behavior is relational, dynamic and multi-dimensional, and to measure it, in any analysis, we must consider all of these aspects.

Normally, psychological researchers are interested in self-reported behaviors during experiments, but this raises an important question: Do people behave coherently with respect to what they self-report? This problem probably is more significant than measurement, since it can affect the validity of all of our research. Technologies can be great instruments in psychological investigations by reducing the gap between the individuals' behaviors and their opinions of their behaviors. For example, a test subject could make every effort not be stressed, but the subject actually could be more stressed than he/she thinks and/or more stressed than the general population (i.e., a normative sample). If psychophysiology can be used to understand internal states, behavioral patterns can be used to understand exhibited behaviors (Cipresso, 2015; Krakauer et al., 2017). Exhibited behaviors can also differ from what we expect. For example, in the famous Nisbett and Wilson experiment of 1977 (Nisbett and Wilson, 1977), a group of participants, hearing a continuous unsettling noise while watching a movie, declared to have enjoyed the experience less than others who didn't have that distraction. Nisbett and Wilson showed that the expressed pleasantness levels were the same for the group with the noise and the other group without the noise. This is surprisingly true in psychological experiments and needs to be considered when self-reported measures are quantified. The lesson learned is that exhibited behaviors are not only the expressed behavior, and this needs to be taken into account in behavioral research.

In particular, activity-related behavior suggests an action regulation that is clearly continuous and observable and can be identified in expressions, contours and other qualities, including vocal tonality. Moreover, gesture and posture are important cues of human communication and part of non-verbal behavior representing internal states, even if “unsaid.” On the other hand, verbal behavior could be interpreted to indicate the “said” elements (Nisbett and Wilson, 1977; Giakoumis et al., 2012; Gomez-Marin et al., 2014).

Technologies to detect exhibited behavior also are now available in low-cost devices, such as Microsoft Kinect, which was built for gaming but has been used extensively in behavioral research. High-end technologies also are used for the analysis of behaviors; for example, they are used for path analysis for neurological patients and in motion-capture systems. Other technologies that have been used extensively in behavioral research are based on body movements (such as Kinect or Nintendo Wii) and eye movements (by using commercial and high-end eye-trackers) (Cipresso et al., 2013). More recently, accelerometers and gyroscopes have been used as laboratory devices or in mobile devices we use every day (such as smartphones). Smartphones have become an important tool for researchers who are interested in understanding behavior during daily activities and in the field (out of the laboratory) (Cipresso et al., 2012a; Gaggioli et al., 2013). The complexity of sensors included in current smartphones allows us to know an individual's position (with GPS), phone calls made (and received), physical activity and many other exhibited behaviors. Also, smartphones make it convenient for people to self-report their activities during specific daily contexts.

Other classic technologies used to attain people's exhibited behaviors are audio and video devices, which are used for both qualitative studies and quantitative analysis. Some of these devices can be very sophisticated, providing speech analysis and video analysis for information retrieval based on artificial intelligence (Camastra and Vinciarelli, 2008).

## Virtual reality

One of the best ways to experience situations in a controlled environment, such as in a laboratory, is definitively Virtual Reality (VR). Thanks to VR, researchers are able to understand and measure several cognitive and emotional states and traits, and even personality traits (Heim, 1993; Ryan, 2001; Sherman and Craig, 2002; Cipresso and Serino, 2014; Villani et al., 2016; Kane and Parsons, 2017). From a technological perspective, VR requires a standard commercial PC capable of 3D visualization, a head-mounted display (HMD) endowed with position trackers and a game controller, such as a joypad (Riva et al., 2015). The image changes in real time thanks to the information that the tracker through the computer records from the position and orientation of the users' HMD in the subjects' head.

Psychologists usually describe VR as “an advanced form of human-computer interface that allows the user to interact with and become immersed in a computer-generated environment in a naturalistic fashion” (Schultheis and Rizzo, 2001). In general, the sense of *presence*, i.e., the feeling of being “inside” the simulated world, is the key element of VR as a communication device (Riva et al., 2014, 2015). In the same way that individuals are consciously “being there,” the feeling of presence in a technologically mediated environment provides a very similar experience; i.e., subjects are not “outside” the synthetic experience, but are “inside” it. In other words, VR provides an experience to be lived as if in a real place, at the same time allowing experimental control in a lab setting with a totally manipulated environment (Riva et al., 2015).

In the past, the use of VR was limited by the expensive cost of the hardware device and software licenses. Over the last few years, the huge market of different head-mounted displays (HMDs), such as Oculus, HTC VIVE, OSVR, Gear, and others, has made it possible to have a bundle of a PC and VR system, including HMD, joypad and other input devices, for < 3,000 Euro (Brooks, 1999; Riva et al., 2003; Riva and Waterworth, 2014). However, unfortunately, the cost of the software is still problematic, not for the licenses, but for the personnel costs for making an integrated VR requiring code knowledge that psychologists are not prone to learn. In this effort, Riva and colleagues tried in the last decades to create open access and free solutions for creating virtual environments without the need of any code (Riva et al., 2011; Cipresso and Riva, 2016).

In the ‘90s, cybertherapy and virtual rehabilitation were considered interesting fields of research with several challenges and problems to solve (Lamson, 1994; North et al., 1996; Rothbaum et al., 1997). However, several clinical, controlled trials demonstrated the efficacy of cybertherapy (Riva, 2003, 2005; Holden, 2005; Malloy and Milling, 2010; McCann et al., 2014). The contemporary reducing of the costs of HMD led to a new era of cybertherapy and virtual rehabilitation, making the field of interest of assessment and quantitative measurement also in the clinical field.

Since VR is a computer-based program, it can track everything. This is a great benefit for quantitative psychology and measurement since the researcher is able to have precise, per-millisecond data for each event that occurs during the experience. Thus, VR can elicit behavior in a replicable setting and simultaneously is able to record data and computing indexes by keeping experimental conditions as well. The use of VR allows the building of complex settings within which researchers can manipulate and replicate to test realistic situations for the behavioral aspects, reported to be relational, dynamic and multi-dimensional by definition (Cipresso, 2015). VR also can be connected to external devices and, within virtual environments, it is possible to integrate devices, such as biosensors, making it possible to measure quantitatively the experience during navigation by using specific interconnected biosensors and internal logs that provide indications about each event (Cipresso, 2015).

By fusing data from biosensors and devices interconnected within the VR environments, it is possible to synchronize all these signals with the log of the VR events that the researcher has set to identify experimental conditions as well as unexpected occurrences, incidental findings and all of the behaviors one may wish to analyze. In this sense, VR can be considered a great way to collect quantitative data of people's actual behaviors during realistic situations in simulated environments.

## Computational science, complex networks, and simulations

One of the most pervasive scientific paradigms in the twenty-first century has been complexity. From hard science to social science, the use of such a paradigm affected the evolution of how we think about given phenomena. The idea that very simple interacting elements are able to produce a complex structure is fascinating, but it also is useful, since it allows us to explain complex phenomena as having emerged from the interactions of simple elements that can be understood and analyzed (Miller and Page, 2009).

The bottom-up approach exploits complexity, opens new ways to study psychological constructs, and provides new tools to answer old questions. Indeed, psychology historically experienced and contributed to the diffusion of complexity, with superb contributions in artificial intelligence, complex networks, psychophysics, and, in general, by using the theoretical and pragmatic level models, methods, and concepts that still are part of complex science (Myung, 2000; Friedenberg, 2009; Guastello et al., 2009).

The increased computational capacity that is currently available provides a new approach to quantitative psychology, and, more generally for measurements, to think well beyond just the new way to analyze data. For example, network complexity is used extensively to analyze relational data, but it also is used to create new ways of thinking in psychology, such as the network theory of mental disorders (Fried and Cramer, 2016; Jones et al., 2017). In addition, computational technologies are providing new ways to create psychological platforms for the assessment of patients in clinical settings as well as their rehabilitation. The use of mobile applications, virtual reality, and psychophysiology for psychological science is also becoming even more computationally oriented by also integrating classification, automatic recognition, and machine-learning algorithms for measurements, as well as for use as a new way to treat mental disorders (Villani et al., 2011, 2016; Michalski et al., 2013; Serino et al., 2013; Cipresso et al., 2017).

From this perspective, pervasiveness and unobtrusiveness are the keys for the integration of computational technologies and methods in psychological tools.

## Toward the challenges of the twenty-second century

**Figure 1:**
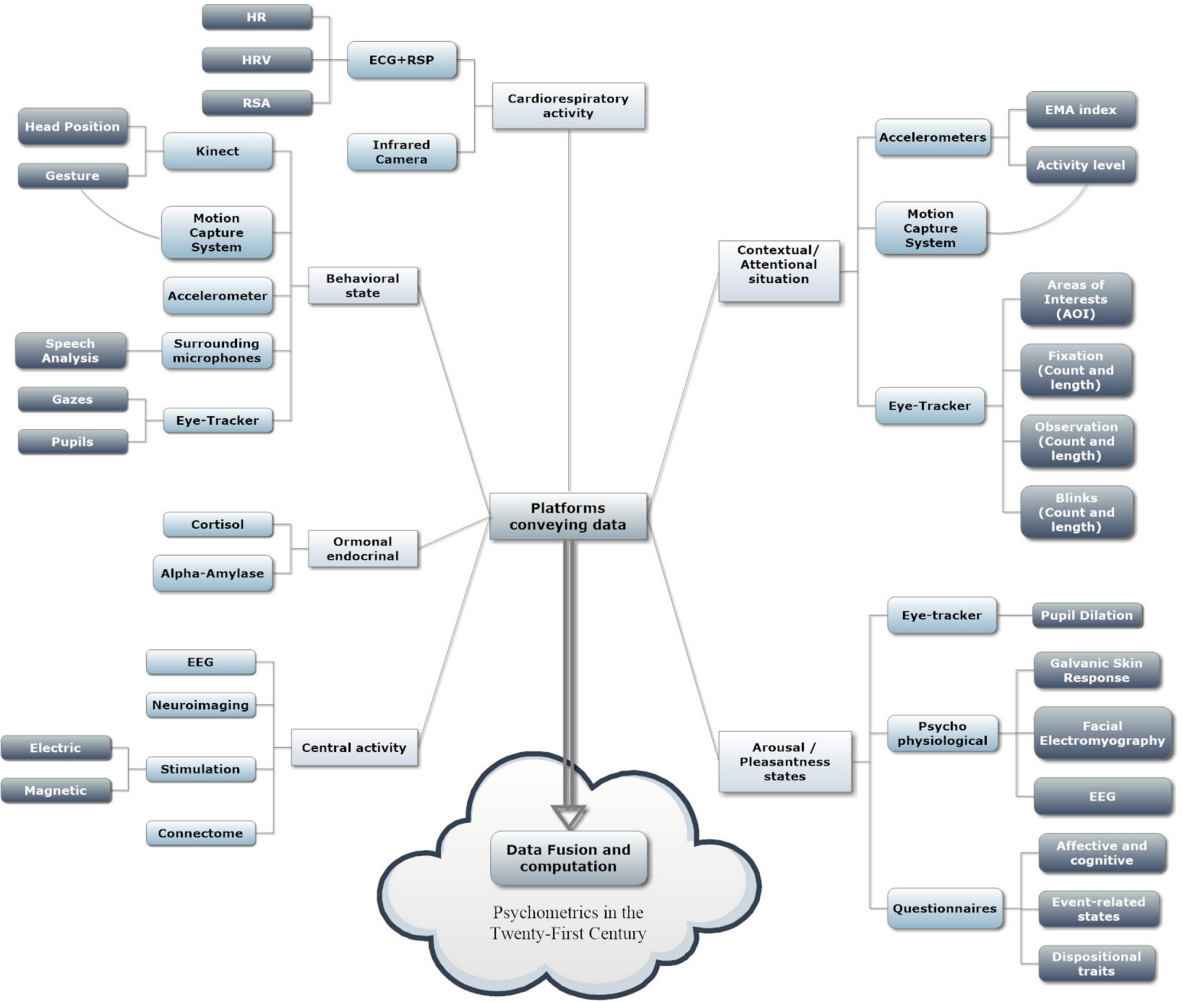
Data fusion and computation from the available sources.

Considering the current development of psychometric tools for quantitative psychology and measurement, we posit that the first two decades of the twenty-first century highlighted a future in which human-computer confluence was possible at the methodological and practical levels (Figure 1). It seems clear that neuropsychological assessment and psychological evaluation will be based on technologies and computational methods, but we can expect more than this for the future. The pervasiveness of low-cost and high-end technologies is exploding, and we can expect that, in the next few decades, they will be integrated further into our daily lives and into objects (e.g., the Internet of Things, IoT). They will be so unobtrusive as to be invisible to the users, e.g., contactless biosensors that can record physiological signals without any patches or sensors on the body (acting from a distance or with conductive objects, such as a chair that record the patient's ECG from her or his back or the use of a mouse to determine the conductance level of human skin) (Cipresso et al., 2013; Cipresso, 2015).

Further, we can expect the use of many other technologies and methods. For example, we cannot exclude a “personalized psychology,” such as the well-known “personalized medicine,” to use genetic information for the understanding of functional and dysfunctional behavior.

In any case, the use of new technologies and new methods can only be driven by new psychologists, in particular new psychometricians who rely on the actual knowledge of psychological science as it is at the moment, but also can build new ways of thinking about psychological settings, experiments, studies, and, above all, interventions. These capabilities will provide a deeper understanding of human behavior and lead to improvements in the well-being of humankind.

## Author contributions

PC and JI conceived the idea. PC wrote the manuscript. PC and JI revised the manuscript and approved the final version.

### Conflict of interest statement

The authors declare that the research was conducted in the absence of any commercial or financial relationships that could be construed as a potential conflict of interest.
